# Remote homology identification of the *Drosophila melanogaster* ortholog of the RNA Polymerase I subunit Rpa34/POLR1G

**DOI:** 10.17912/micropub.biology.001107

**Published:** 2024-01-12

**Authors:** Ryan Palumbo, Bruce Knutson

**Affiliations:** 1 Department of Biochemistry & Molecular Biology, SUNY Upstate Medical University, Syracuse, New York, United States

## Abstract

Highly conserved orthologous proteins are easily identified by sequence homology alone, whereas poorly conserved orthologs require additional structural information to be identified. All *Drosophila* orthologs of RNA polymerase I, II, and III subunits—except one—have been identified by sequence homology. Here, we identified CG11076 as the missing Rpa34/POLR1G ortholog in *Drosophila* . Remote homology detection and secondary structure analysis showed that CG11076 is predicted to have high structural conservation with Rpa34/POLR1G, and phylogenetic analysis demonstrated that these proteins are closely related. Our work underscores the importance of utilizing both sequence and structure to identify highly divergent orthologous proteins in different species.

**Figure 1. CG11076 is the Drosophila ortholog of yeast Rpa34 and human POLR1G:**
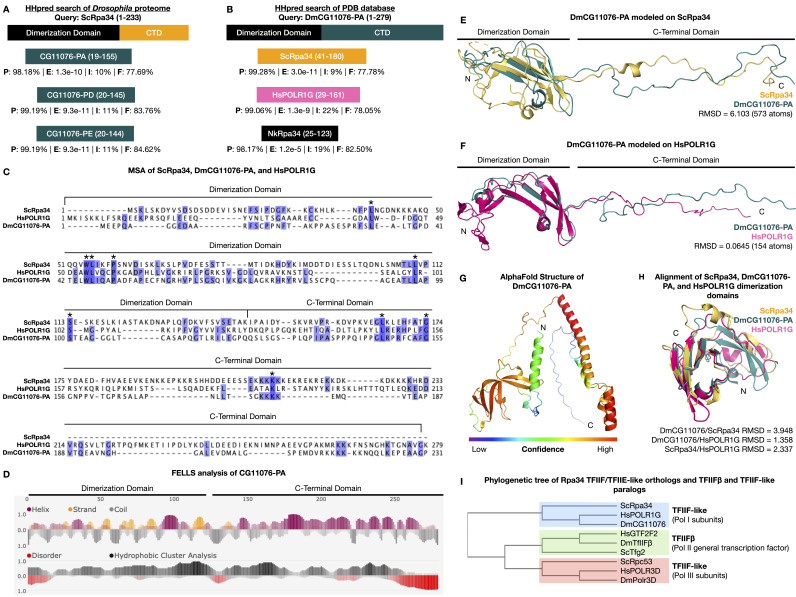
(A-B) HHpred results for *S. cerevisiae* Rpa34 (ScRpa34) against the *Drosophila* proteome (A) and *Drosophila* CG11076-PA (DmCG11076-PA) against the PDB database (B). Topmost bar represents the domain structure of the query protein. CTD: carboxy-terminal domain. The bottom three bars represent the target proteins. Target bars overlap the region of the query that was aligned by HHpred. P: probability (the likelihood of a positive match between the query and target), E: E-value (the average number of nonhomologous hits expected), I: percent identity (percent of identical residues between the query and target), F: percent fold (the percent of equivalent query and target residues predicted to have a similar secondary structure). (C) Multi Sequence Alignment (MSA) of ScRpa34, human POLR1G (HsPOLR1G), and DmCG11076-PA using HHpred. Residues highlighted in blue are identical in at least two sequences. Residues that are identical in all three sequences are marked by an asterisk. Note that the long, disordered C-terminal domain of HsPOLR1G was not included in the MSA to prevent erroneous gaps in the MSA. (D) FELLS secondary structure analysis and disorder prediction of DmCG11076-PA. (E-F) DmCG11076-PA modeled onto the Cryo-EM structures of ScRpa34 (E) and HsPOLR1G (F) using MODELLER. Alignment and RMSD calculations were made in PyMol. (G) AlphaFold predicted structure of DmCG11076-PA, colored by confidence (b-factor) in PyMol. (H) Alignment of the beta sheet segments of the AlphaFold predicted structures of ScRpa34, DmCG11076-PA, and HsPOLR1G. Alignment and RMSD calculations were made in PyMol. (I) Phylogenetic analysis of Rpa34 orthologs and paralogs from *S. cerevisiae* , *Drosophila* , and human.

## Description

RNA Polymerase I (Pol I) is an essential multiprotein complex required for the synthesis of pre-ribosomal RNA. In the yeast *Saccharomyces cerevisiae* , Pol I is made up of 14 subunits, whereas in humans, Pol I is made up of only 13 subunits. Rpa34 and Rpa49 in *S. cerevisiae* , and POLR1G and POLR1E in humans, are Pol I subunits that form a TFIIF/TFIIE-like heterodimer through a conserved beta barrel dimerization domain. This heterodimer functions at many steps throughout the transcription cycle (Geiger et al. 2010; Vannini and Cramer 2012; Knutson et al. 2020) . Although Rpa34 and POLR1G are conserved in *S. cerevisiae* and humans, the fruit fly *Drosophila melanogaster* lacks an identifiable ortholog of Rpa34 based on sequence homology (Marygold et al. 2020) . This is surprising because the *Drosophila* genome encodes an ortholog of the Rpa34 dimerization partner, Rpa49 (Polr1E). It is unlikely that one protein from this heterodimer would be lost from the *Drosophila* genome, therefore, we hypothesized that *Drosophila* does encode an ortholog of Rpa34/POLR1G, but that it has diverged considerably such that sequence homology alone cannot detect it. To overcome this, we employed remote homology detection that considers structural information along with sequence homology to increase the sensitivity and depth of our search.

To search for a *Drosophila* ortholog of *S. cerevisiae* Rpa34, we used HHpred (Söding 2005; Hildebrand et al. 2009; Meier and Söding 2015; Zimmermann et al. 2018; Gabler et al. 2020). HHpred is a powerful and highly sensitive remote homology search method that readily detects homology between distantly related proteins, or those that have diverged considerably in sequence, as we predicted was the case for a *Drosophila* Rpa34 ortholog. The protein sequence of Rpa34 was used to query the *Drosophila* proteome, and the only three matches with high probability and significant E-values corresponded to three isoforms of uncharacterized *Drosophila* protein CG11076 ( Figure 1A ). The top two matches were identical isoforms of CG11076: CG11076-PD and CG11076-PE. Each isoform comprises 260 amino acids and appear to be expressed from the same promoter, but with different transcription start sites (TSS; FlyBase.org). The third match was CG11076-PA, which is identical to CG11076-PD and CG11076-PE, except for an additional 19 amino acids at the N-terminus. CG11076-PA is expressed from a different promoter with a distinct TSS.

Sequence identity between the CG11076 isoforms and Rpa34 was low (Figures 1A, 1C). There was only 10-11% identity between the residues of the CG11076 isoforms that aligned to Rpa34. Alternatively, we considered the percent of aligned residues predicted to have a similar fold (i.e., coil or random coil structure, helix structure, beta strand or extended conformation, or isolated beta bridge residue). In contrast with sequence identity, the percent of aligned residues predicted to have a similar fold was high, ranging from 77.69% to 84.62% ( Figure 1A ). These results suggest that CG11076, the putative *Drosophila* ortholog of *S. cerevisiae* Rpa34, has strong secondary structure conservation with Rpa34, but very little sequence identity. The lack of sequence identity between CG11076 and Rpa34 likely accounts for why previous work to identify *Drosophila* Pol subunits via sequence homology was unable to identify a *Drosophila* ortholog of *S. cerevisiae* Rpa34 (Marygold et al. 2020) .

Given that CG11076 and Rpa34 have similar predicted secondary structure features ( Figure 1A ), we next compared the predicted secondary structure of CG11076 to experimentally determined structures of Rpa34 orthologs. To do this, we used the sequence of CG11076-PA (the canonical UniProt isoform) to query the PDB database using HHpred (Söding 2005; Hildebrand et al. 2009; Meier and Söding 2015; Zimmermann et al. 2018; Gabler et al. 2020). The only three matches with high probability and significant E-values all corresponded to Rpa34 orthologs ( Figure 1B ). The top match was *S. cerevisiae* Rpa34, from the Cryo-EM structure of the *S. cerevisiae* Pol I preinitiation complex (PDB 6RUI, chain N; Sadian et al. 2019). The second match was the human ortholog of *S. cerevisiae* Rpa34, POLR1G, from the Cryo-EM structure of elongating human Pol I (PDB 7VBB, chain N; Zhao et al. 2021). The third match was the *Nakaseomyces glabratus* (the yeast formerly known as *Candida glabrata* ) ortholog of Rpa34, from the crystal structure of the Rpa34/Rpa49 heterodimer (PDB 3NFG, chain B; Geiger et al. 2010). Again, the sequence identity between CG11076 and the orthologs was low (Figures 1B, 1C). There was only 9-22% identity between the residues of CG11076-PA that aligned to *S. cerevisiae* Rpa34, human POLR1G, or *N. glabratus* Rpa34. In contrast with sequence identity, the percent of aligned residues predicted to have a similar fold was very high, ranging from 77.78% to 82.50%. The results suggest that the predicted secondary structure of CG11076 is like those of the experimentally determined structures of three different Rpa34 orthologs.

Both *S. cerevisiae* Rpa34 and human POLR1G consist of an N-terminal TFIIF/TFIIE-like beta barrel dimerization domain followed by a disordered C-terminal domain (CTD; Sadian et al. 2019; Zhao et al. 2021). To determine whether CG11076-PA follows a similar structural synteny, we analyzed the secondary structure of CG11076-PA using FELLS (Piovesan et al. 2017) . Secondary structure analysis of CG11076-PA ( Figure 1D ) showed that the N-terminus is enriched in beta sheet segments, as expected for a TFIIF-like beta barrel domain. Likewise, the CG11076-PA C-terminal domain, particularly near the last third of the CTD, lacked high confidence secondary structure and was predicted to be disordered like its *S. cerevisiae* and human orthologs. These observations are also supported by the AlphaFold structure of CG11076-PA, which predicted that the N-terminus of CG11076 comprises beta sheet segments, but that the very end of the CTD is highly disordered ( Figure 1G ).

To further assess the structural similarity between CG11076-PA and either *S. cerevisiae* Rpa34 or human POLR1G, the protein sequence of CG11076-PA was modeled onto the Cryo-EM structures of *S. cerevisiae* Rpa34 and human POLR1G using MODELLER (Šali et al. 1995; Webb and Sali 2016; Zimmermann et al. 2018; Gabler et al. 2020). The resulting structures of CG11076-PA were aligned to *S. cerevisiae* Rpa34 ( Figure 1E ) and human POLR1G ( Figure 1F ) using PyMol. We also used PyMol to calculate the root mean square deviation (RMSD) for each alignment, which assesses the similarity between the aligned structures. While the RMSD of the CG11076-PA/Rpa34 alignment was 6.103, the RMSD for the CG11076-PA/POLR1G alignment was 0.645. These RMSD values indicate that model of CG11076 aligns better to human POLR1G than to *S. cerevisiae* Rpa34. This is expected, given that *Drosophila* and human are both metazoans and are more closely related than either is to *S. cerevisiae* . Nevertheless, both CG11076-PA models aligned well to either the *S. cerevisiae* Rpa34 or human POLR1G Cryo-EM structures, especially within the highly conserved N-terminal beta sheets that make up the dimerization domain. Moreover, the N-terminus of CG11076-PA is predicted with high confidence by AlphaFold to form beta sheet segments ( Figure 1G ). Therefore, we aligned the beta sheet regions from the AlphaFold structures of *S. cerevisiae* Rpa34 and human POLR1G to that of CG11076-PA to determine whether the predicted structures also aligned well ( Figure 1H ). The RMSD value for the CG11076/Rpa34 alignment was 3.948 (154 atoms), for the CG11076/POLR1G alignment was 1.358 (199 atoms), and for the Rpa34/POLR1G alignment was 2.337 (326 atoms). Overall, these results suggest strong structural conservation between *Drosophila* CG11076 and human POLR1G.

*S. cerevisiae* Rpa34 (TFIIF-like subunit of Pol I) is a paralog of both *S. cerevisiae* Tfg2 (subunit of Pol II general transcription factor TFIIFβ) and *S. cerevisiae* Rpc53 (TFIIF-like subunit of Pol III). *Drosophila* TfIIFβ and human GFT2F2 are homologous to *S. cerevisiae* Tfg2, and *Drosophila* Polr3D and human POLR3D are homologous to *S. cerevisiae* Rpc53. To determine the evolutionary relationships between CG11076 and these Rpa34 orthologs/paralogs in Pols I, II, and III, we constructed an unrooted phylogenetic tree from multiple HHPred sequence alignments encompassing the conserved beta barrel dimerization domains of these proteins ( Figure 1I ). As expected, CG11076 clusters with *S cerevisiae* Rpa34 and human POLR1G (i.e., its Pol I counterparts), demonstrating that they are evolutionarily related.

Together, our *in silico* analysis of the structural properties of *Drosophila* CG11076 in comparison with those of *S. cerevisiae* Rpa34 and human POLR1G suggest strong structural homology between the three proteins. Therefore, we propose that CG11076 is the missing *Drosophila* ortholog of Rpa34/POLR1G, bringing the total number of *Drosophila* Pol I subunits to 13, as in humans. Following the naming convention of Marygold et al. (2020), we suggest hereon referring to CG11076 as Polr1G.

## Methods

The protein sequence of *Saccharomyces cerevisiae* Rpa34 (UniProt P47006, release 2023_05) was used to query the entire *Drosophila* proteome (Euk_Drosophila_melanogaster_19_Jul_2017) using HHpred ( https://toolkit.tuebingen.mpg.de/tools/hhpred ; Söding 2005; Hildebrand et al. 2009; Meier and Söding 2015; Zimmermann et al. 2018; Gabler et al. 2020). The protein sequence of CG11076-PA (the canonical UniProt isoform, Q9V493, release 2023_05) was used to query the PDB_mmCIF70_4_Oct structural/domain database using HHpred. The protein sequences of CG11076-PA (UniProt Q9V493, release 2023_05), *S. cerevisiae* Rpa34 (PDB 6RUI, chain N; Sadian et al. 2019), and human POLR1G (PDB 7VBB, chain N, lacking the C-terminal domain, for clarity; Zhao et al. 2021) were aligned using HHpred, and colored by percent identity in Jalview (Waterhouse et al. 2009) . The secondary structure and predicted disorder of CG11076-PA were analyzed and displayed using the FELLS server with default settings ( http://old.protein.bio.unipd.it/fells/ ; Piovesan et al. 2017). The protein sequence of CG11076-PA was modeled onto *S. cerevisiae* Rpa34 using MODELLER ( https://toolkit.tuebingen.mpg.de/tools/modeller ; Šali et al. 1995; Zimmermann et al. 2018; Gabler et al. 2020), and the resulting structure of CG11076-PA was aligned to *S. cerevisiae* Rpa34 using PyMol (via the “super” alignment method). The protein sequence of CG11076-PA was modeled onto human POLR1G using MODELLER, and the resulting structure of CG11076-PA was aligned to human POLR1G using PyMol [via the “align” alignment function with outlier rejection (cycles: 5, cutoff: 2.0)]. To determine the structural conservation between CG11076, *S. cerevisiae* Rpa34, and human POLR1G, the N-terminal beta sheet segments from the AlphaFold structures of Rpa34 (AF-P47006-F1-model_v4, residues 32-142), CG11076-PA (AF-Q9V493-F1-model_v4, residues 40-122), and POLR1G (AF-O15446-F1-model_v4, residues 21-120) were reciprocally aligned using PyMol [via the “align” alignment function in PyMol with outlier rejection (cycles: 5, cutoff: 2.0)]. This produced RMSD values for each pairwise alignment. To align and perform phylogenetic analysis of TFIIF-like and TFIIFβ subunits, the *Drosophila* and human orthologs of *S. cerevisiae* Tfg2 and Rpc53 (paralogs of Rpa34) were identified using the FlyBase Homolog Search tool (FlyBase.org, release FB2023_06). *Drosophila* TfIIFβ (UniProt P41900, release 2023_05) and human GFT2F2 (UniProt P13984, release 2023_05) are homologous to *S. cerevisiae* Tfg2 (UniProt P41896, release 2023_05), and *Drosophila* Polr3D (UniProt Q8SYM9, release 2023_05) and human POLR3D (UniProt P05423, release 2023_05) are homologous to *S. cerevisiae* Rpc53 (UniProt P25441, release 2023_05). HHpred was used to produce multiple sequence alignments (MSA) of the conserved beta barrel dimerization domains of *S. cerevisiae, Drosophila* , and human Rpa34 orthologs/paralogs. These alignments were combined and converted to FASTA format. Phylogenetic analysis and tree construction was performed using the Simple Phylogeny program ( https://www.ebi.ac.uk/Tools/phylogeny/simple_phylogeny/ ; Madeira et al. 2022). Default settings were used, except for using a NEXUS tree format and the UPGMA clustering method. The resulting tree was viewed with a cladogram branch length.
